# An Integrated Deep Learning and Belief Rule Base Intelligent System to Predict Survival of COVID-19 Patient under Uncertainty

**DOI:** 10.1007/s12559-021-09978-8

**Published:** 2021-12-16

**Authors:** Tawsin Uddin Ahmed, Mohammad Newaj Jamil, Mohammad Shahadat Hossain, Raihan Ul Islam, Karl Andersson

**Affiliations:** 1 Department of Computer Science and Engineering, University of Chittagong, Chittagong, Bangladesh; 2 Pervasive and Mobile Computing Laboratory, Luleå University of Technology, S-931 87 Skellefteå, Sweden

**Keywords:** COVID-19, Transfer Learning, VGG Net, Validation Accuracy, Belief Rule Base

## Abstract

The novel Coronavirus-induced disease COVID-19 is the biggest threat to human health at the present time, and due to the transmission ability of this virus via its conveyor, it is spreading rapidly in almost every corner of the globe. The unification of medical and IT experts is required to bring this outbreak under control. In this research, an integration of both data and knowledge-driven approaches in a single framework is proposed to assess the survival probability of a COVID-19 patient. Several neural networks pre-trained models: Xception, InceptionResNetV2, and VGG Net, are trained on X-ray images of COVID-19 patients to distinguish between critical and non-critical patients. This prediction result, along with eight other significant risk factors associated with COVID-19 patients, is analyzed with a knowledge-driven belief rule-based expert system which forms a probability of survival for that particular patient. The reliability of the proposed integrated system has been tested by using real patient data and compared with expert opinion, where the performance of the system is found promising.

## Introduction

The sudden appearance of an unknown member of a large virus family is not a novel experience for humans. Almost every century in human history, viruses with novel genome sequences appear and take thousands of lives. Ebola, Swine Flu, SARS, HIV, Hong Kong Flu, and Asian Flu are the deadliest viruses that caused the death of a large number of lives. In December 2019, an unrecognized viral pneumonia patient got identified in Wuhan, China. It is the city where the novel Coronavirus is discovered first. Anyway, Severe Acute Respiratory Syndrome (SARS) Coronavirus (COV)-2 belongs to the Coronavirus family, which causes mild-to-moderate respiratory symptoms and five to ten percent of infected people show severe respiratory symptoms [1]. This novel Coronavirus is responsible for a respiratory disease, addressed as Coronavirus Disease 2019 or COVID-19. It is a highly contagious disease, transferred from one person to another by droplets, direct contact with the infected individual, or touching an infected object. Due to its massive transmission capability, it has already spread all over the globe within a few months. Moreover, this Coronavirus outbreak put the health sectors of most countries at risk, and the World Health Organisation (WHO) has announced it as a pandemic [2].

Deep learning approaches are vastly adopted in solving computer vision problems [3, 4]. In medical imaging, deep learning methods are also integrated into numerous medical equipment to diagnose critical diseases. X-ray, CT scan, MRI, etc., contain health information of a patient, which are useful input parameters for deep learning models [5]. Automated information extraction from medical images by neural nets makes diagnosis faster for medical professionals. Transfer learning is one of the most remarkable concepts in deep learning [6]. It refers to the loading of learned weights from a pre-trained model without training a model from scratch. Transfer learning is a proper choice if a researcher has a limited dataset, which is the case for COVID-19 research. Coronavirus attacks the epithelial cells that line the respiratory tract, which can be identified from chest X-ray images.

Belief Rule-Based Expert System (BRBES) is an expert system driven by the extension of traditional IF-THEN rules where the system delivers a consequence value with a degree of belief [7]. This consequence value of a rule is constructed based on its antecedent attributes with referential values. In this research, BRBES makes decisions by analyzing CNN-generated linguistic output along with some crucial risk factors regarding a patient’s health. With these inputs, BRBES performs input transformation, rule activation weight calculation, belief update, rules aggregation using evidential reasoning, and finally generates patient survival probability.

The core contributions and significance of this research can be addressed as follows: Unlike the typical COVID-19 patient detection from X-ray images, the proposed integrated framework can contribute to the survival probability assessment of COVID-19 patients. It helps medical professionals to adopt immediate measurements in critical situation. In the process of health condition analysis of the COVID-19 infected individuals, this research takes into consideration both data-driven deep learning and knowledge-driven Belief Rule-Based approach. Total dependency on data-driven or knowledge-driven schemes is excluded in this research which encourages the reliability in patient’s health condition assessment. In addition to the patient condition assessment, a novel dataset COVID-19-Status (X-ray images of critical and non-critical patients) is also proposed in this research which is made public for further research in this domain.

The remainder of this article is structured as follows: short briefs on several related works are provided in Related Work. Convolutional Neural Network and Data Collection and Augmentation demonstrate the mechanism of the Convolutional Neural Network and data collection and augmentation, while Belief Rule-Based Expert System provides a short description of the Belief Rule-Based Expert System. Then Integration of CNN and BRBES depicts how the integration of CNN and BRBES takes place in this research, and Experiment provides details about experiments. Demonstration of the system implementation and result analysis is delivered in System Implementation and Result and Discussion. Finally, Conclusion concludes the article.

## Related Work

Sousa et al. [8] aim to find out what factors are linked to COVID-19 death rate and recovery in a state in Brazil’s northeast. Only moderate and severe cases were hospitalized according to a survey on patients who had flu-like symptoms, sought medical help, and tested COVID positive till April 2020. Robust Poisson regression was used to evaluate mortality, while Kaplan–Meier and Cox regression was used to investigate survival. For two thousand seventy COVID-19 patients, the survival rate is 87.7% considering from the twenty-fourth day of infection. The clinical risk factors taken into account in this research as the parameter for assessing the survival probability are diabetes, CVD, hematologic disorders, pneumopathies, immunodeficiencies, neurological diseases, asthma and so on.

Dong et al. [9] investigated that among the admitted COVID-19 sufferers, higher neutrophil-to-lymphocyte ratio and NT-proBNP readings and hypertension are linked with a worse outcome. LASSO and multivariate Cox regression models are taken into account in the training phase to find predictive markers for hospitalized COVID-19 patients’ survival. For medical usage, a nomogram characterized by three factors is formulated. In model training and testing cohorts, AUCs and C-index are utilized to assess the nomogram’s performance. In the train and test batches, the nomogram’s C-indices are 0.901 and 0.892, correspondingly. In the learning phase, the area under curve for fourteen and the twenty-one-day likelihood of hospitalized COVID-19 survival are 0.922 and 0.919, accordingly, whereas, in the test batch, it shows 0.922 and 0.881.

The goal of Murillo-Zamora et al. [10] was to investigate the medical criteria and risk factors for deaths associated with COVID-19, which is actively circulating in the population in Mexico. By investigating the risk factors, the researchers found that diabetes, obesity, chronic obstructive pulmonary disease, chronic kidney disease, smoking habit, hypertension, and even age and sex are all significantly linked to the chance of death, especially for COVID-19 patients. This research examined 331,298 COVID-19 diagnosed patients to see whether factors are linked to death. The probabilities of death of features and morbidities in COVID-19 patients were studied using multivariate logistic regression and Kaplan–Meier survival curves.

Nemati et al. [11] experimented with various learning algorithms to examine the mortality factors of nearly twelve hundred patients in this study. Several machine-learning (gradient boosting, SVM), as well as statistical analysis (CoxPH, Coxnet, KM estimator), are incorporated to assess the release prognosis of COVID-19 patients. Only a minimal number of parameters, such as age, sex, available dates, and status (death or release), are preserved in the dataset among the original one where the major part of the data comes from public healthcare reports and online resources, which are mostly provided by state/local medical authorities and clinics in various nations. The results show that the gradient boosting model surpasses alternative algorithms in this investigation for predicting patient mortality.

COVID-19 data from two hundred and eighty-seven patients admitted in Saudi Arabia’s King Fahad University Hospital are analyzed Aljameel et al. [12] in this research. Classification techniques are adopted to examine the data: random forest, extreme gradient boosting, logistic regression. Several preprocessing approaches are employed in order to prepare the data at first. In addition, 10-K-fold cross-validation and SMOTE are incorporated to fragment the samples and ameliorate the imbalance among them. Investigations are carried out with 20 clinical variables that are found to be relevant in predicting survival versus death in COVID-19 patients. With an efficiency of 0.95 and an AUC of 0.99, the findings indicate that RF surpassed the remaining predictors. The proposed model can successfully aid decision-making and medical practitioners by immediate diagnosis of critical COVID-19 patients.

The articles discussed above assess the survival probability of the COVID-19 patient formulated based on some risk factors. These risk factors’ involvement is equally significant as the chest radiography analysis, which indicates to what extent the lungs get infected of a COVID-19 patient. This parameter is also crucial to consider, especially for COVID-19 patients, because this disease results in lung damage in critical situations. In this research, most possible factors associated with a COVID-19 patient are taken into account and assess the probability outcome regarding the patient’s mortality.

## Convolutional Neural Network

Chest X-ray images of COVID-19 patients are considered to be analyzed to assess their condition using a deep learning approach. Varying chest X-ray image values correspond to several density groups. For example, the dark area of the X-ray images refers to the space covered with air, whereas the off-white portion indicates the descend tissue (bones). In the case of lungs, which is likely to be affected for critical COVID-19 patients with pneumonia, the air-filled dark portion of the lungs appears to be dense due to liquid-like substances in the X-ray images, which refer to pulmonary abnormalities. Moreover, the cost-effectiveness and availability of X-ray technology over computed tomography (CT scan) technology is one of the reasons to analyze X-ray images.

The transfer learning approach is adopted in this research rather than building the CNN model from scratch. The CNNs frameworks of pre-trained models are already loaded with trained weight so that they are already familiar with the features of an image. Several pre-trained CNN models are taken into account in this X-ray image classification task and make a comparison among them. Xception, InceptionResNetV2, VGG16, and VGG19 models are considered to be trained on a chest X-ray image dataset and select an appropriate model that can detect whether or not a COVID-19 patient is in critical condition based on the patient’s chest X-ray image. All the mentioned pre-trained CNN models are previously trained on one of the largest image datasets in computer vision, “ImageNet” which contains more than 14 million images distributed among more than 2000 classes [13]. After applying these pre-trained models on the X-ray image dataset, a performance comparison is drawn to propose the optimum model for chest X-ray image classification. It should be mentioned that selected pre-trained models are gone through some fine-tuning mechanisms in the fully connected layers rather than applying exactly the same pre-trained model architecture in order to improve classification accuracy.

**Fig. 1:**
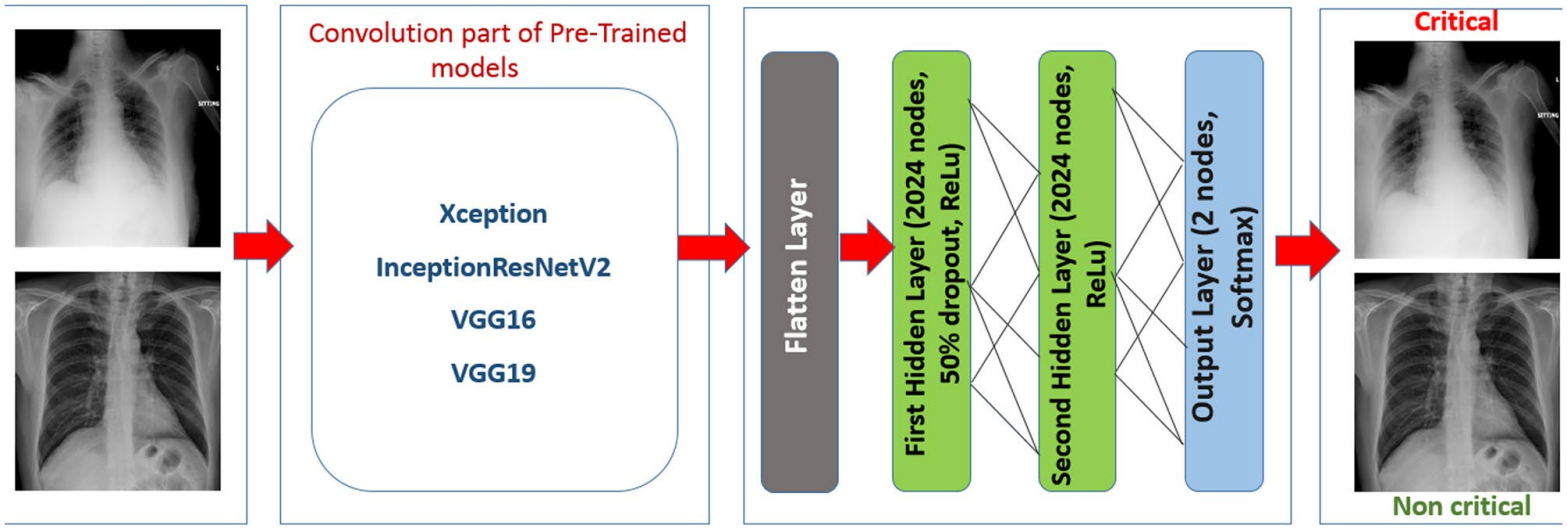
Schematic Representation of the Research Plan

According to Fig. 1, which represents the schematic representation of the research plan, dataset images are fed into the fine-tuned pre-trained models. In the tuning process, fully connected layers are customized by including two hidden layers following the flatten layer. Each of the hidden layers consists of 2024 nodes. Moreover, in between the hidden layers dropout layer of 0.5 is introduced to prevent the issues regarding model overfitting [14]. The dropout layer takes the responsibility that the model does not get biased to training data and delivers better prediction performance on the validation data. Although models have already trained on a large “ImageNet” dataset and are acquainted with image features, it requires dataset-specific features to get better recognition performance. In order to fulfil this purpose, instead of freezing all layers, some convolution, batch normalization, or pooling layers of the base model are included in the training phase so that these layers can extract more dataset-specific features from the chest X-ray image dataset. Table 1 shows the layers that are selected for training and other than these layers, the remaining layers are kept excluded for training.

**Table 1:** Trainable Layers of the Pre-trained Models

**Pre-Trained Models**	**Trainable Layers**
Xception	2 Convolution layers, 2 Batch normalization layers
InceptionResNetV2	4 Convolution layers, 2 Batch normalization layers
VGG16	6 Convolution layers, 2 MaxPooling layers
VGG19	6 Convolution layers, 2 MaxPooling layers

According to Table 1, only the last layers weights of the pre-trained models are updated during back-propagation which enables a reduction in computation time. The motive of freezing initial layers is that these models are already trained on huge dataset like ImageNet [44] and so they are well-acknowledged with lower-level basic image features like edges and curves. Now, later convolution layers of the proposed model are responsible for acquiring data-specific high-level features. Although the total number of layers Xception, InceptionResNetV2, VGG16, and VGG19 have is 36, 164, 16, and 19, respectively, in this research maximum of eight layers are included in the training process.

## Data Collection and Augmentation

Cohen et al. [15] introduce a COVID-19 chest X-ray image dataset and make it available to a GitHub repository. Most of the research works conducted on COVID-19 approve and use this standard dataset. Since it is a novel topic of research, data collection is still going on and the number of images of the repository is expanding as days pass by. Till the time when this research was being conducted, the dataset contained a total of 673 images that were unequally distributed among nine different classes. The number of images per class is: COVID-19 (538), Streptococcus (17), Pneumocystis (17), SARS (16), Pneumonia (14), Mycoplasma Bacterial Pneumonia (8), Klebsiella (8), Legionella (6), Lipoid (5), Varicella (5), Bacterial (4), E.Coli (4), ARDS (4), Chlamydophila (2), and Influenza (2). The remaining 23 images are unlabeled. In order to prepare the dataset for this research, the chest X-ray images of the COVID-19 class are closely observed. It should be mentioned that COVID-19 causes severe pneumonia at the critical condition of the patient and on that note, X-ray images at the initial stage and critical stage of COVID-19 patients are differentiable.

**Fig. 2:**
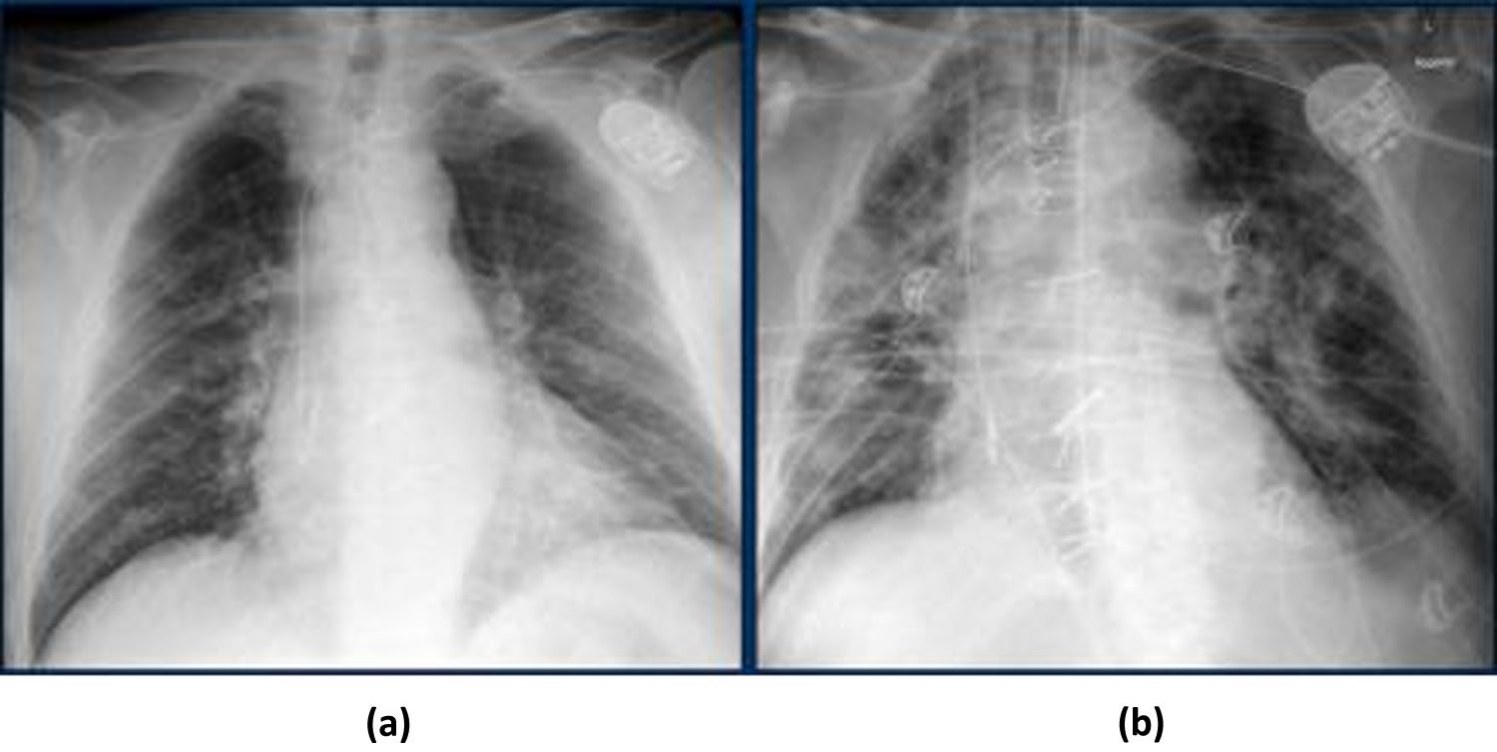
X-ray Images of COVID-19 Patient in (**a**) Non-critical and (**b**) Critical Condition

If Fig. 2 is observed, according to [16], these X-rays are of a patient with COVID-19. On admission to the hospital, the chest condition was normal, which is the initial stage of COVID-19 in Fig. 2(a). But after four days, the patient is on ventilation, and there were bilateral consolidations on the chest X-ray in Fig. 2(b) [16]. Based on this, 673 X-ray images of COVID-19 patients are labeled with critical and non-critical classes keeping apart the other classes of the original dataset. After labeling, the “Critical” class consists of 148 images and 390 images are included in the “Non-critical” class. But still not enough data for the deep learning model that is why the data augmentation method is applied to the existing dataset. The reason for using data augmentation is the dataset of the original source narrowed down and especially the “Critical” class has only 148 images. And the characteristic of the data-driven approach like CNN is that it maintains a positive correlation, in terms of prediction capability, with the size of the dataset. Traditional image augmentation techniques like horizontal flip, rotation, shear, and zoom are incorporated in this research. Random clockwise image rotation with 30% rotation degree and shifting and zooming image by 20% are applied to generate additional data. The parameters that are considered in the data augmentation method are provided in Table 2 with value.

**Table 2:** Data Augmentation Parameters with Value

**Operation Type**	**Value**
Horizontal Flip	True
Rotation	0.30
Shear	0.20
Zoom	0.20

Although augmented images are generated from the existing image dataset, variance in parameter allows the augmentation method to construct images of different view angles. This image variation defends the possibility of model overfitting.

**Fig. 3:**
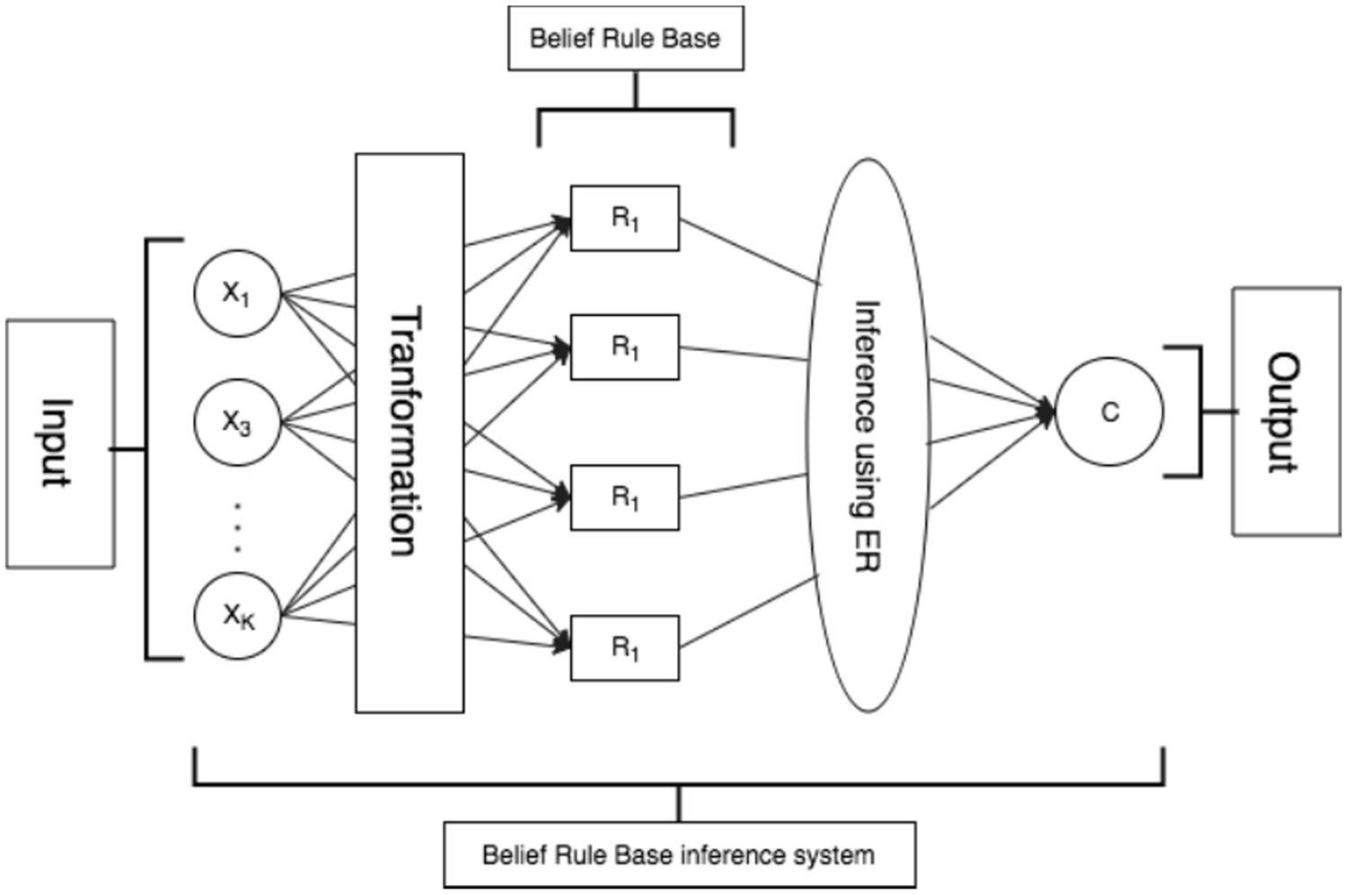
Sample Images of COVID-19-Status [17] Dataset

So, a derived “COVID-19-Status” dataset is proposed in this research which is made available in GitHub [17]. Figure 3 shows sample images of the COVID-19-Status dataset.

## Belief Rule-Based Expert System

A Belief Rule-Based Expert System (BRBES) is composed of two main components [18]. The first one is Belief Rule Base (BRB), which works as a knowledge base, and the second one is evidential reasoning that works as an inference engine [19]. By extending the traditional IF-THEN rule, a belief rule is formed with two main parts. The first part is a collection of antecedent attributes, which are linked with referential values, and the second part is the consequent attribute that is embedded with belief degrees. In a BRB, attribute weight, rule weight, and belief degrees are the knowledge representation parameters, which are accountable to capture uncertainty in data [20]. A belief rule is presented below:

$$\begin{aligned} R_k:{\left\{ \begin{array}{ll} \text {IF Patient Condition is Critical AND Blood Pressure is Elevated} \\ \text {AND Chronic Obstructive Pulmonary Disease is Mildly Abnormal} \\ \text {AND Blood Sugar is Normal AND Asthma is Intermittent} \\ \text {AND Chronic Kidney Disease is Very Severe AND Obesity is Level II} \\ \text {AND Acute Respiratory Distress Syndrome is Mild} \\ \text {AND Pulse Oxymetry is Moderate} \\ \text{THEN Patient Survival Probability is} \\ \text {(Very High,0.0), (High,0.0), (Medium, 0.55), (Low, 0.45), (Very Low, 0.0)} \end{array}\right. } \end{aligned}$$

In this rule, “Patient Condition”, “Blood Pressure”, “Chronic Obstructive Pulmonary Disease”, “Blood Sugar”, “Asthma”, “Chronic Kidney Disease”, “Obesity”, “Acute Respiratory Distress Syndrome”, and “Pulse Oxymetry” are the antecedent attributes, while ‘Critical’, ‘Elevated’, ‘Mildly Abnormal’, ‘Normal’, ‘Intermittent’, ‘Very Severe’, ‘Level II’, ‘Mild’, and ‘Moderate’ are their corresponding referential values. “Patient Survival Probability” is the consequent attribute and its referential values are ‘Very High’, ‘High’, ‘Medium’, ‘Low’, and ‘Very Low’. The belief distribution of this consequent attribute is (Very High, 0.0), (High, 0.0), (Medium, 0.55), (Low, 0.45), and (Very Low, 0.0). In this rule, the sum of belief degrees (0.0 + 0.0 + 0.55 + 0.45 + 0.0 = 1) associated with each referential values of the consequent attribute is one, so the rule is complete. However, the rule is considered incomplete if the sum of belief degrees is less than one, which can happen due to ignorance or incompleteness.

**Fig. 4:**
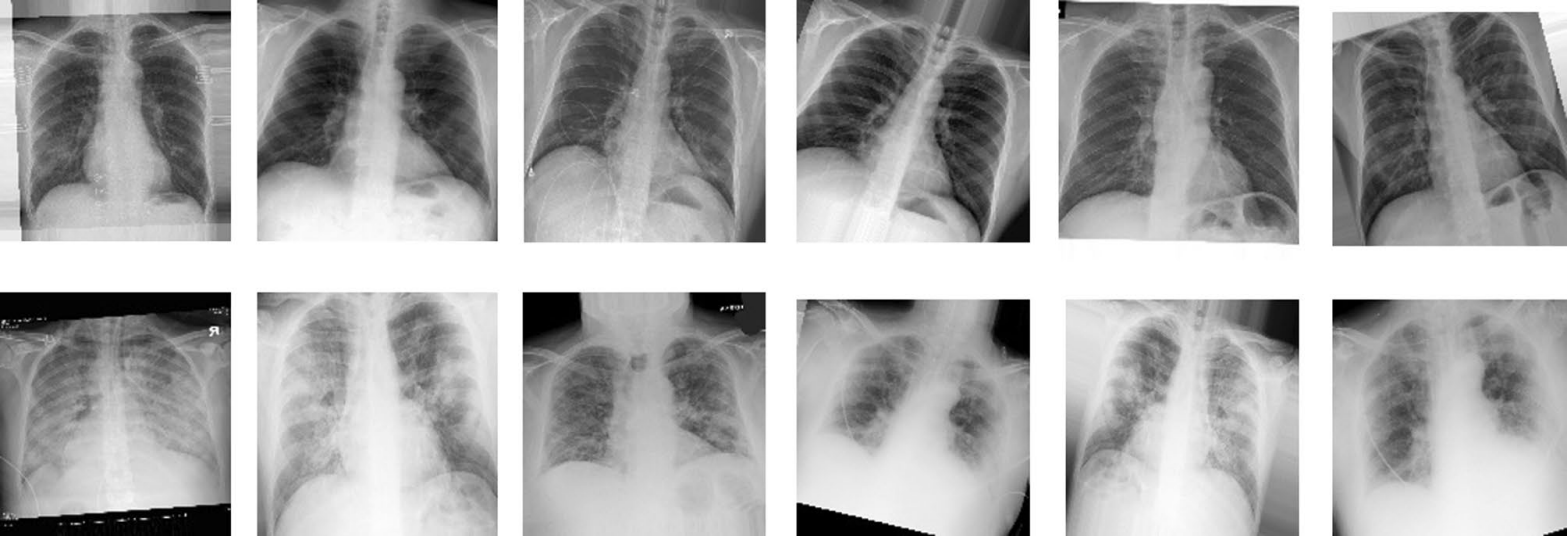
Sequence of BRBES Inference Procedures

Evidential reasoning (ER) can handle heterogeneous data and different types of uncertainties, including incompleteness, ignorance, imprecision, and vagueness. It consists of four steps, namely input transformation, rule activation weight calculation, belief update, and rule aggregation, which is shown in Fig. 4.

The first step is input transformation, where the input data are distributed over the referential values of the antecedent attributes of a belief rule, which is known as matching degrees [21]. After calculating matching degrees, the belief rules are called packet antecedent, and they are considered active [22]. The matching degrees are used to perform the second step, which is the calculation of the activation weight of the rules. The third step is belief update, where the belief degree associated with each belief rule in the rule base is updated in case of ignorance or missing input data for any of the antecedent attribute [23]. The fourth step is rule aggregation, which is performed by using either analytical or recursive evidential reasoning algorithm [24]. The crisp value is calculated from the fuzzy output of the rule aggregation procedure using the utility score associated with each referential value of the consequent attribute [25]. All these steps are performed by following the procedures mentioned in [26, 27].

## Integration of CNN and BRBES

**Fig. 5:**
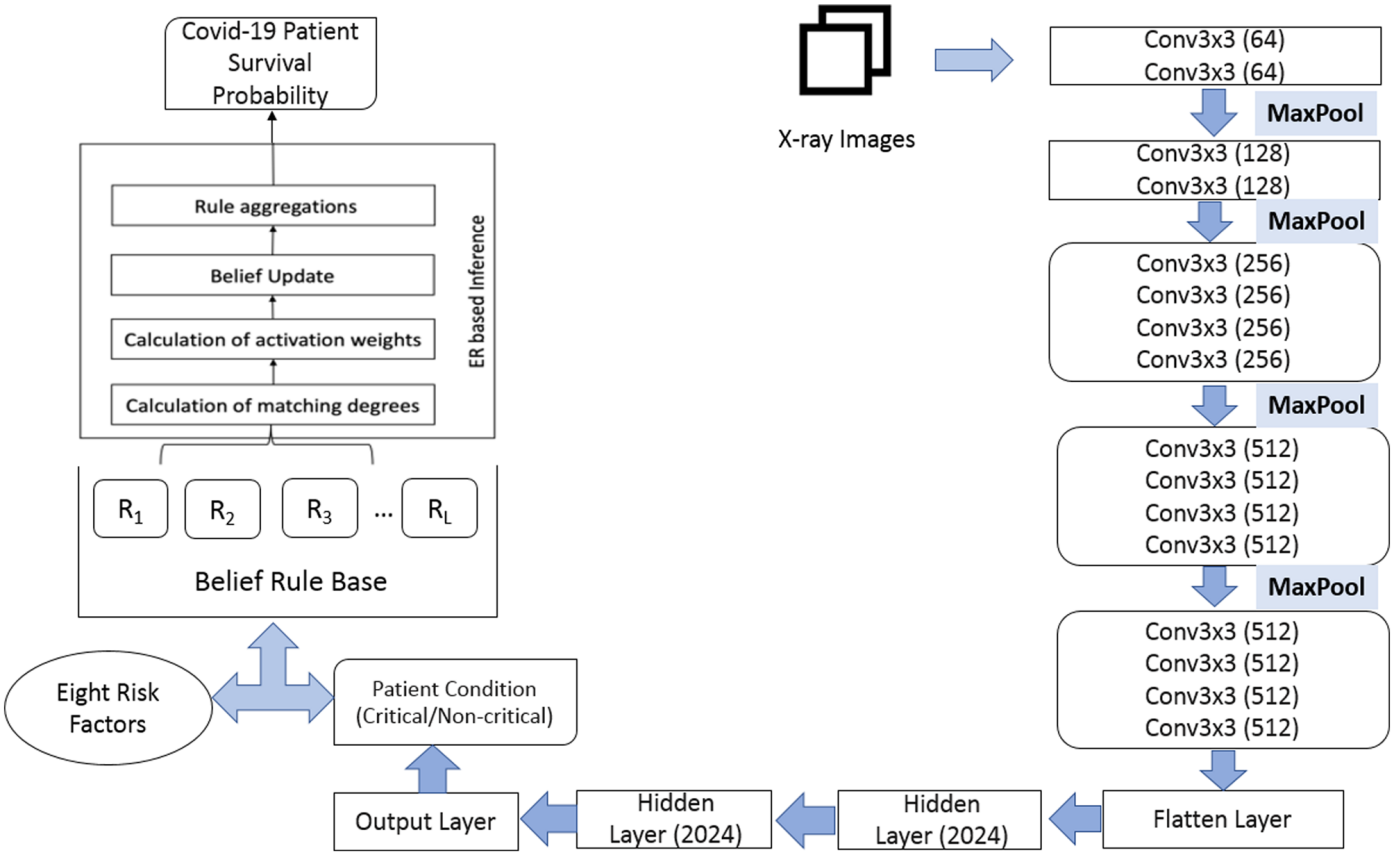
The Workflow of the Proposed CNN-BRBES Integrated Framework

Both Convolutional Neural Network (CNN) and Belief Rule-Based Expert System (BRBES) are incorporated in this research to assess the survival probability of a COVID-19 patient based on X-ray image along with considering several risk factor parameters of that patient. The step-by-step workflow of the proposed integrated framework is depicted in Fig. 5.

- As mentioned earlier, the pre-trained model VGG19 is optimized in this X-ray image classification task by making some changes in its original architecture based on observed experimental results. This change is made basically in the fully connected layer portion. However, the convolution portion is kept as it is, which consists of 16 convolution layers with 3x3 convolution filter size. Four MaxPooling layers contribute to extract the feature-max in between the convolution layers. Instead of using 4096 nodes per hidden layer, 2024 nodes are included with 50% dropout in each of the two hidden layers. VGG19 architecture ends with two output layers for critical and non-critical classes.
- Now, the CNN part of the integrated approach is responsible for X-ray image analysis to classify “Patient Condition” into one of the two categories. Basically, it imitates the image analysis methodology of the human brain and recognizes the class of that image. Trainable convolution layers of CNN are accountable for the extraction of high-level features of an X-ray image and generate a feature map for each filter. Feature vectors are basically two-dimension feature information that is converted to a one-dimension vector so that fully connected layer performs weight adjustment using backpropagation. With the help of the Softmax activation function, probabilities are distributed among the classes. Then class with higher probability is converted to a linguistic form, which is either ‘Non-Critical’ or ‘Critical’ and delivered to BRBES as one of the input parameters.
- Other input parameters that are considered for BRBES are some crucial risk factors of that patient such as “Blood Pressure”, “Chronic Obstructive Pulmonary Disease”, “Blood Sugar”, “Asthma”, “Chronic Kidney Disease”, “Obesity”, “Acute Respiratory Distress Syndrome”, and “Pulse Oxymetry” [28–31]. Usually, patients and physicians use linguistic terms to express these risk factors. For example, “Chronic Obstructive Pulmonary Disease” is expressed by using linguistic terms such as ‘Normal’, ‘Mildly Abnormal’, ‘Moderately Abnormal’, ‘Severely Abnormal’, and ‘Very Severely Abnormal’.
- Therefore, a numerical scale has been considered to convert linguistic terms into a numerical value. For example, “Very Severely Abnormal” is given the preference value as “10”, “Severely Abnormal” is given the preference value as “8”, “Moderately Abnormal” is given the preference value as “5”, “Mildly Abnormal” is given the preference value as “2” and “Normal” is given the preference value as “0”. Table 3 shows the numerical scale of measurement for each input parameter for BRBES.
- Based on these input parameters, “Patient Survival probability” is calculated using BRBES. For “Patient Survival probability”, its referential values are chosen as ‘Very High’, ‘High’, ‘Medium’, ‘Low’, and ‘Very Low’, and its utility values are chosen as ‘100’, ‘75’, ‘50’, ‘25’, and ‘0’, respectively. It is worth mentioning that all the risk factors, their linguistic measurement categories, and corresponding numerical scaling are determined under the supervision of clinical experts.

In general, there are two types of Belief Rule Base (BRB), namely conjunctive BRB and disjunctive BRB. In conjunctive BRB, each rule is assumed as conjunctive in nature, while in disjunctive BRB, each rule is represented using disjunctive assumption.

**Table 3:** Numerical Scale of Measurement for Each Input Parameter for BRBES

**Symptom**	**Linguistic Term**	**Numerical Scale**
Patient condition	Critical	10
	Non Critical	1
Blood pressure	Hypertensive Crisis (Systolic: > 180 mm Hg and/or Diastolic: > 120 mm Hg)	10
	Stage 2 Hypertension (Systolic: $\ge$ 140 mm Hg or Diastolic: $\ge$ 90 mm Hg)	8
	Stage 1 Hypertension (Systolic: 130-139 mm Hg or Diastolic: 80-89 mm Hg)	5
	Elevated (Systolic: 120-129 mm Hg and Diastolic: < 80 mm Hg)	2
	Normal (Systolic: < 120 mm Hg and Diastolic: < 80 mm Hg)	0
Chronic obstructive pulmonary disease	Very Severely Abnormal (FEV-1:$\le$30%)	10
	Severely Abnormal (FEV-1: 30-49%)	8
	Moderately Abnormal (FEV-1: 50-69%)	5
	Mildly Abnormal (FEV-1: 70-79%)	2
	Normal (FEV-1:$\ge$80%)	0
Blood sugar	Diabetic (Fasting:$\ge$125 mg/dL and Post Meal:$\ge$200 mg/dL)	10
	Pre-Diabetic (Fasting: 101-125 mg/dL and Post Meal: 141-200 mg/dL)	5
	Normal (Fasting: 70-100 mg/dL and Post Meal: 70-140 mg/dL)	0
Asthma	Severe Persistent (Symptoms: Throughout the day)	10
	Moderate Persistent (Symptoms: Daily)	8
	Mild Persistent (Symptoms: > 2 days per week, but not daily)	5
	Intermittent (Symptoms:$\le$2 days per week)	2
	Normal (No Symptoms)	0
Chronic kidney disease	Very Severe (GFR: < 15 mL/min)	10
	Severe (GFR: 15-29 mL/min)	8
	Moderate (GFR: 30-59 mL/min)	5
	Mild (GFR: 60-89 mL/min)	2
	Normal (GFR: > 90 mL/min)	0
Obesity	Level III (BMI:$\ge$40)	10
	Level II (BMI: 35-39.9)	6
	Level I (BMI: 30-34.9)	3
	Normal (BMI: < 30)	0
Acute respiratory distress syndrome	Severe (PaO2/FiO2:$<=$100 mmHg)	10
	Moderate (100 mmHg < PaO2/FiO2:$<=$200 mmHg)	6
	Mild (200 mmHg < PaO2/FiO2:$<=$300 mmHg)	3
	Normal (PaO2/FiO2: > 300 mmHg)	0
Pulse oximetry	Severe (Saturation:$<=$93%)	10
	Moderate (Saturation: > 93%)	1

Under the conjunctive assumption, the total number of rules, L, is calculated using the referential values, $J_i$ of the antecedent attributes, $A_i$ of a BRB, as shown in Eq. (1).

1 $$\begin{aligned} L = \prod _{i=1}^{T_k} J_i \end{aligned}$$

Under the disjunctive assumption, the total number of rules, L, is equal to the number of referential values of the antecedent attributes, as shown in Eq. (2). The disjunctive assumption requires that all attributes have the same number of referential values [32].

2 $$\begin{aligned} L = J_1 = J_2 = ... = J_i \end{aligned}$$

As mentioned above, nine input parameters have been considered for BRBES to calculate patient survival probability. From Table 3, it can be seen that each input parameter has a different number of referential values. Hence, it is not possible to consider disjunctive BRB. Therefore, conjunctive BRB has been considered for BRBES, where the total number of rules = $2\times 5\times 5\times 3\times 5\times 5\times 4\times 4\times 2=120,000$.

Usually, a BRB can be established in four ways, namely by extracting belief rules from domain expert knowledge, extracting belief rules by examining historical data, using previous rule bases if available, and using random rules if there is no prior knowledge [33]. In this study, due to the lack of prior knowledge, the initial BRB is constructed by using random rules as follows. First, intermediate values within the range of consequence values have been calculated. Then the number of possible combinations has been calculated using the length of each referential value. Finally, after calculating intermediate values for each combination, the belief degree associated with each referential value of the consequent attribute has been distributed within the range.

The initial BRB for patient survival probability is shown in Table 4 which consists of rule id with corresponding rule weight, input antecedents from X1 to X9 and their respective inferred consequence in terms of survival probabilities of the patients. For better representation, all the input parameters are assigned to one-to-one variable X where X1: Patient Condition, X2: Blood Pressure, X3: Chronic Obstructive Pulmonary Disease, X4: Blood Sugar, X5: Asthma, X6: Chronic Kidney Disease, X7: Obesity, X8: Acute Respiratory Distress Syndrome and X9: Pulse Oximetry. The categories for the severity of the risk factors are denoted as Critical: C, Non-Critical: NC, Hypertensive Crisis: HC, Very Severely Abnormal: VSA, Diabetic: D, Severe Persistent: SP, Very Severe: VS, Level III: L-III, Severe: S, Moderate: M, Normal: N, Mild: Mi, VH: Very High, H: High, M: Medium, L: Low, VL: Very Low. And the survival probability of the COVID-19 patients taking into account the risk factors and X-ray images is denoted by Y.

**Table 4:** Initial Belief Rule Base for Patient Survival Probability: Y (X1: Patient Condition, X2: Blood Pressure, X3: Chronic Obstructive Pulmonary Disease, X4: Blood Sugar, X5: Asthma, X6: Chronic Kidney Disease, X7: Obesity, X8: Acute Respiratory Distress Syndrome, X9: Pulse Oximetry, Critical: C, Non Critical: NC, Hypertensive Crisis: HC, Very Severely Abnormal: VSA, Diabetic: D, Severe Persistent: SP, Very Severe: VS, Level III: L-III, Severe: S, Moderate: M, Normal: N, Mild: Mi, VH: Very High, H: High, M: Medium, L: Low, VL: Very Low)

Rule	Rule	IF									THEN (*Y*)				
ID	Weight	*X*1	*X*2	*X*3	*X*4	*X*5	*X*6	*X*7	*X*8	*X*9	VH	H	M	L	VL
1	1	C	HC	VSA	D	SP	VS	L-III	S	S	1	0	0	0	0
2	1	C	HC	VSA	D	SP	VS	L-III	S	M	0.59	0.41	0	0	0
3	1	C	HC	VSA	D	SP	VS	L-III	M	S	0.82	0.18	0	0	0
4	1	C	HC	VSA	D	SP	VS	L-III	M	M	0.41	0.59	0	0	0
...	...	...	...	...	...	...	...	...	...	...	...	...	...	...	...
119997	1	NC	N	N	N	N	N	N	Mi	S	0	0	0	0.55	0.45
119998	1	NC	N	N	N	N	N	N	Mi	M	0	0	0	0.14	0.86
119999	1	NC	N	N	N	N	N	N	N	S	0	0	0	0.41	0.59
120000	1	NC	N	N	N	N	N	N	N	M	0	0	0	0	1

In order to ensure the reliability of the rules in the initial BRB, the BRBES is trained using the non-linear optimization solver *fmincon* in MATLAB optimization toolbox [34], Belief Rule-Based Adaptive Particle Swarm Optimization (BRBAPSO) [35], and the enhanced Belief Rule-Based adaptive Differential Evolution (eBRBaDE) algorithm [36]. The detailed procedure to train the BRBES can be found in [34–36]. After training the BRBES using *fmincon*, BRBAPSO, and eBRBaDE, the trained BRB for patient survival probability is shown in Tables 5, 6, and 7, respectively. Various rule weights for the rules can be observed from Tables 5, 6, and 7. For example, 0.89 has been set as rule weight for the rule 1 of *fmincon* trained Belief Rule Base, whereas 0.94 and 0.98 for rule weight for the rule 1 BRBAPSO and eBRBaDE trained Belief Rule Base, respectively. The reference values of the consequent attributes for the rule 1 are (0.77,0.23,0,0) of the *fmincon* trained Belief Rule Base. The reference values of the consequent attributes for the rule 1 are (0.83,0.17,0,0) and (0.87,0.13,0,0) for BRBAPSO and eBRBaDE trained Belief Rule Base, respectively. Here, it can be observed that meta-heuristic-based algorithm like BRBAPSO and eBRBaDE are predicting similar values in comparison to deterministic algorithm like *fmincon*

**Table 5:** Trained Belief Rule Base Using *fmincon* for Patient Survival Probability (Y) where input antecedents are X1: Patient Condition, X2: Blood Pressure, X3: Chronic Obstructive Pulmonary Disease, X4: Blood Sugar, X5: Asthma, X6: Chronic Kidney Disease, X7: Obesity, X8: Acute Respiratory Distress Syndrome, X9: Pulse Oximetry, Critical: C, Non Critical: NC, Hypertensive Crisis: HC, Very Severely Abnormal: VSA, Diabetic: D, Severe Persistent: SP, Very Severe: VS, Level III: L-III, Severe: S, Moderate: M, Normal: N, Mild: Mi, VH: Very High, H: High, M: Medium, L: Low, VL: Very Low)

Rule	Rule	IF									THEN (*Y*)				
ID	Weight	*X*1	*X*2	*X*3	*X*4	*X*5	*X*6	*X*7	*X*8	*X*9	VH	H	M	L	VL
1	0.89	C	HC	VSA	D	SP	VS	L-III	S	S	0.77	0.23	0	0	0
2	0.78	C	HC	VSA	D	SP	VS	L-III	S	M	0.42	0.58	0	0	0
3	0.71	C	HC	VSA	D	SP	VS	L-III	M	S	0.59	0.41	0	0	0
4	0.74	C	HC	VSA	D	SP	VS	L-III	M	M	0.26	0.74	0	0	0
...	...	...	...	...	...	...	...	...	...	...	...	...	...	...	...
119997	0.68	NC	N	N	N	N	N	N	Mi	S	0	0	0	0.41	0.59
119998	0.79	NC	N	N	N	N	N	N	Mi	M	0	0	0	0.14	0.86
119999	0.75	NC	N	N	N	N	N	N	N	S	0	0	0	0.36	0.64
120000	0.85	NC	N	N	N	N	N	N	N	M	0	0	0	0.06	0.94

**Table 6:** Trained Belief Rule Base Using BRBAPSO for Patient Survival Probability (Y) where input antecedents are X1: Patient Condition, X2: Blood Pressure, X3: Chronic Obstructive Pulmonary Disease, X4: Blood Sugar, X5: Asthma, X6: Chronic Kidney Disease, X7: Obesity, X8: Acute Respiratory Distress Syndrome, X9: Pulse Oximetry, Critical: C, Non Critical: NC, Hypertensive Crisis: HC, Very Severely Abnormal: VSA, Diabetic: D, Severe Persistent: SP, Very Severe: VS, Level III: L-III, Severe: S, Moderate: M, Normal: N, Mild: Mi, VH: Very High, H: High, M: Medium, L: Low, VL: Very Low)

Rule	Rule	IF									THEN (*Y*)				
ID	Weight	*X*1	*X*2	*X*3	*X*4	*X*5	*X*6	*X*7	*X*8	*X*9	VH	H	M	L	VL
1	0.94	C	HC	VSA	D	SP	VS	L-III	S	S	0.83	0.17	0	0	0
2	0.83	C	HC	VSA	D	SP	VS	L-III	S	M	0.49	0.51	0	0	0
3	0.77	C	HC	VSA	D	SP	VS	L-III	M	S	0.65	0.35	0	0	0
4	0.80	C	HC	VSA	D	SP	VS	L-III	M	M	0.33	0.67	0	0	0
...	...	...	...	...	...	...	...	...	...	...	...	...	...	...	...
119997	0.74	NC	N	N	N	N	N	N	Mi	S	0	0	0	0.47	0.53
119998	0.85	NC	N	N	N	N	N	N	Mi	M	0	0	0	0.21	0.79
119999	0.81	NC	N	N	N	N	N	N	N	S	0	0	0	0.43	0.57
120000	0.94	NC	N	N	N	N	N	N	N	M	0	0	0	0.11	0.89

**Table 7:** Trained Belief Rule Base Using eBRBaDE for Patient Survival Probability (Y) where input antecedents are X1: Patient Condition, X2: Blood Pressure, X3: Chronic Obstructive Pulmonary Disease, X4: Blood Sugar, X5: Asthma, X6: Chronic Kidney Disease, X7: Obesity, X8: Acute Respiratory Distress Syndrome, X9: Pulse Oximetry, Critical: C, Non Critical: NC, Hypertensive Crisis: HC, Very Severely Abnormal: VSA, Diabetic: D, Severe Persistent: SP, Very Severe: VS, Level III: L-III, Severe: S, Moderate: M, Normal: N, Mild: Mi, VH: Very High, H: High, M: Medium, L: Low, VL: Very Low)

Rule	Rule	IF									THEN (*Y*)				
ID	Weight	*X*1	*X*2	*X*3	*X*4	*X*5	*X*6	*X*7	*X*8	*X*9	VH	H	M	L	VL
1	0.98	C	HC	VSA	D	SP	VS	L-III	S	S	0.87	0.13	0	0	0
2	0.89	C	HC	VSA	D	SP	VS	L-III	S	M	0.53	0.47	0	0	0
3	0.83	C	HC	VSA	D	SP	VS	L-III	M	S	0.71	0.29	0	0	0
4	0.87	C	HC	VSA	D	SP	VS	L-III	M	M	0.39	0.61	0	0	0
...	...	...	...	...	...	...	...	...	...	...	...	...	...	...	...
119997	0.79	NC	N	N	N	N	N	N	Mi	S	0	0	0	0.52	0.48
119998	0.91	NC	N	N	N	N	N	N	Mi	M	0	0	0	0.26	0.74
119999	0.88	NC	N	N	N	N	N	N	N	S	0	0	0	0.49	0.51
120000	0.99	NC	N	N	N	N	N	N	N	M	0	0	0	0.14	0.86

In order to show the results of evidential reasoning, an example can be considered. Suppose from the X-ray image analysis of a patient by CNN, the “Patient Condition” is found as ‘Critical’. Suppose the other risk factors of that patient are as follows. Blood Pressure: Elevated, Chronic Obstructive Pulmonary Disease: Mildly Abnormal, Blood Sugar: Normal, Asthma: Intermittent, Chronic Kidney Disease: Moderate, Obesity: Level I, Acute Respiratory Distress Syndrome: Severe, and Pulse Oxymetry: Severe.

Now after performing the four steps of evidential reasoning, namely input transformation, rule activation weight calculation, belief update, and rule aggregation, the “Patient Survival Probability” will be found as: (Very High,0.0), (High,0.0), (Medium, 0.91), (Low, 0.09), (Very Low, 0.0)

Now, from this fuzzy output of the rule aggregation procedure, the crisp value is calculated using the utility score associated with each referential value of the “Patient Survival Probability” attribute as follows.

Patient Survival Probability = $0.0\times 100+0.0\times 75+0.91\times 50+0.09\times 25+0.0\times 0=52.25\% \simeq 52\%$.

**Fig. 6:**
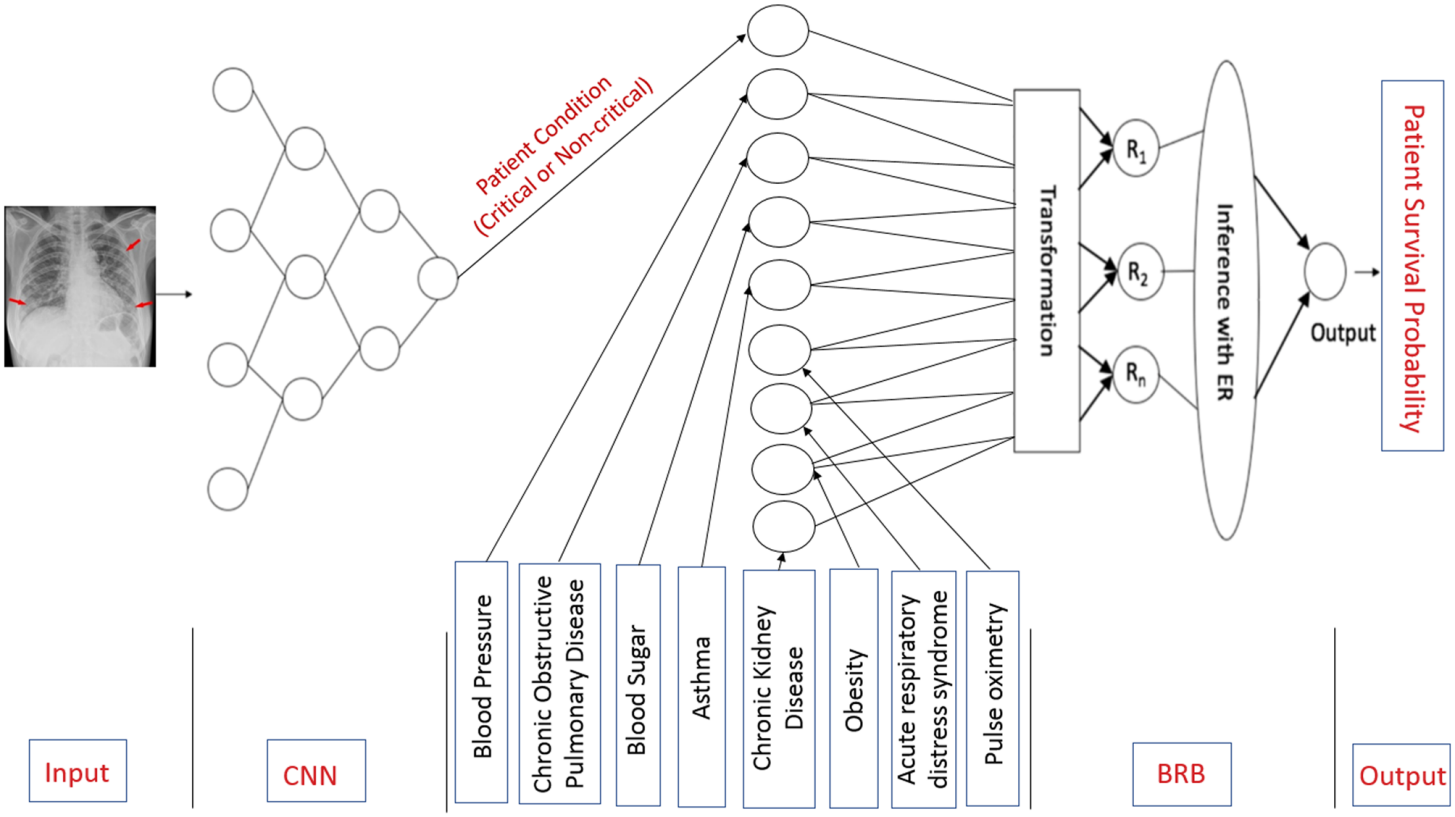
Architecture of Integrated CNN-BRBES System

Finally, the architecture of integrated CNN-BRBES system is shown in Fig. 6.

## Experiment

Several experiments are conducted, especially during fine-tuning the fully connected layers and deciding the number of layers of base pre-trained models that should be added to the training process. As inputs for customized pre-trained models, images with 224x224 dimensions are fed for classification. Each of the image pixels is a combination of 3 channels (red, green, blue). “ImageNet” dataset on which the models are trained contains images of the same pixels and channels. Before data augmentation, the COVID-19-Status dataset [17] had a total of 538 images, with 148 images in critical class and 390 images in non-critical class. However, after augmentation, images are equally distributed between the classes and each class consists of 1122 images that mean the dataset holds a total of 2244 images after data augmentation. Anyway, each of the four models is trained on the Google Colaboratory cloud server, which has shared Tesla K80 GPU [37]. During model training, Adabound optimizer [38] is used because this optimizer is developed combining the positive sides of two popular optimizers: Stochastic Gradient Descent (SGD) and Adam. The learning rate is set to 0.001 in the optimizer. After the completion of experiments, InceptionResNetv2 and VGG19 take 5 epochs, and Xception and VGG16 take 6 epochs to complete their training. In each case, model training is stopped by EarlyStopping callback as there is no improvement in learning after certain epochs.

The dataset splitting ratio of this research is 80:20, which means 80% of the dataset images are selected for model training and 20% is kept for testing purposes.

## System Implementation

**Fig. 7:**
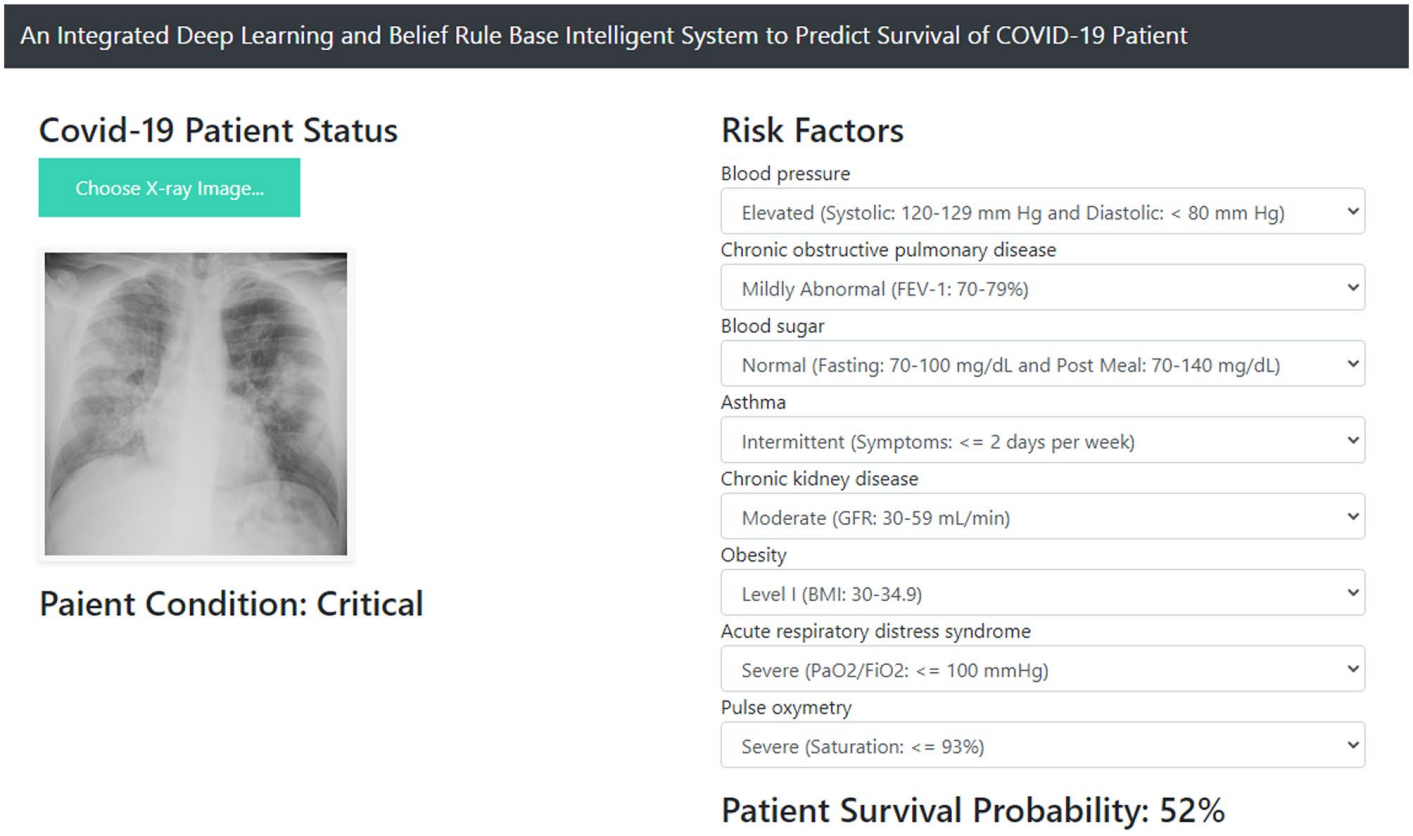
Real Time Validation of the Proposed Model

A Graphical User Interface (GUI) is developed by which users can be facilitated with COVID-19 patient status checking system. To host the model on a server, Flask library of python is used. HTML, CSS, JavaScript are used to design the front-end, and for the server, localhost is employed where 5000 is selected as the port number. This GUI allows a user to upload a chest X-ray image from the local device and this local app resizes the image into 224x224x3. Then with the help of model weight file ‘Covid-19.h5’, it classifies the image and shows the result on the screen just like Fig. 7.

## Result and Discussion

For each model, validation accuracy, recall, precision, and F1 score are considered as model’s performance evaluation parameters.

According to Table 8, the Xception model achieves the lowest validation accuracy and F1 score, which are 83.52% and 83.91%. VGG19 is leading the chart with the highest validation accuracy (99.78%) and F1 score (99.79%) and it has close competition with VGG16, which holds 99.55% and 99.56%, respectively. InceptionResNet50 also provides a firm validation accuracy of 96.21%. All the models achieve these validation accuracies in less than 7 epochs due to their pre-trained weights.

**Table 8:** Performance Evaluation of the Models

**Model Name**	**Validation accuracy (%)**	**Recall (%)**	**Precision (%)**	**F1 score (%)**
Xception	83.52	85.40	82.48	83.91
InceptionResNetV2	96.21	97.35	95.24	96.28
VGG16	99.55	100	99.12	99.56
**VGG19**^**a**^	**99.78**	99.58	**100**	**99.79**

^a^VGG19 has the highest validation accuracy, precision, and F1 score

**Fig. 8:**
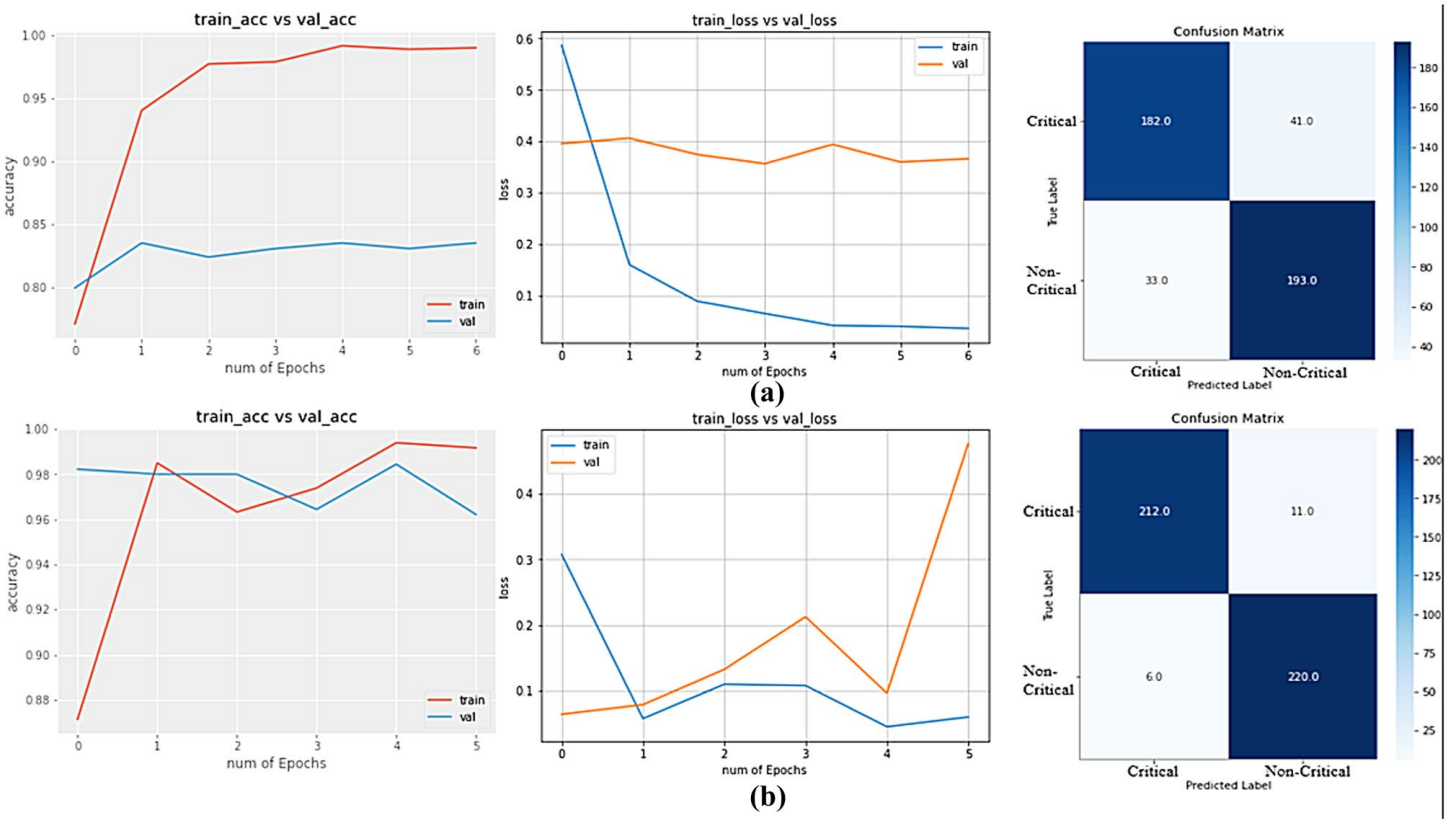
**(a)** Loss and Accuracy Curves, Confusion Matrix of Xception Model; **(b)** Loss and Accuracy Curves, Confusion Matrix of InceptionResNetV2 Model

**Fig. 9:**
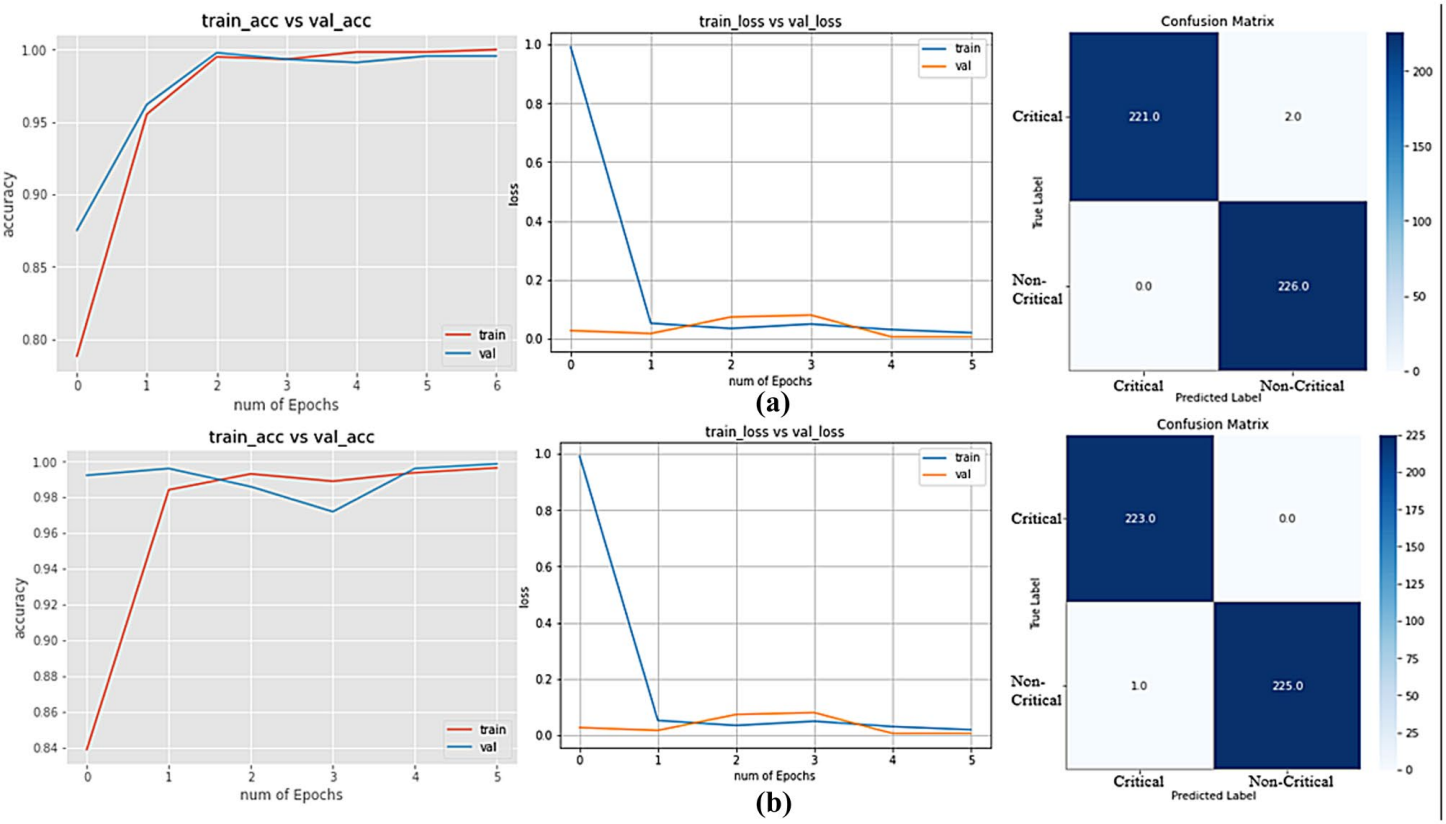
**(a)** Loss and Accuracy Curves, Confusion Matrix of VGG16 Model; **(b)** Loss and Accuracy Curves, Confusion Matrix of VGG19 Model

Figures 8 and 9 depict the accuracy and loss curve of model training and confusion matrix of all applied pre-trained models, where Figs. 8(a, b) and 9(a, b) refer to Xception, InceptionResnetV3, VGG16, and VGG19 model, respectively. From the accuracy curves of the models, it can be claimed that the difference between training and validation accuracy is insignificant except for the Xception model. Xception model shows model overfitting, which means it recognizes the training samples more accurately than validation samples. Other than that, InceptionResNetV2, VGG16, and VGG19 show firm capability of recognizing unseen images. It is commendable that VGG19 recognizes all the unseen images except an image. In the case of VGG16, it fails to recognize only two images.

In order to demonstrate the applicability of the integrated CNN-BRBES, complete data of 200 different COVID-19 patients have been collected with the help of a physician from a hospital located in the Chittagong District of Bangladesh. Among them, 150 patients’ data have been used to train the BRBES using *fmincon* [34], BRBAPSO [35], and eBRBaDE [36], while 50 patients’ data have been used to validate the proposed integrated system. For simplicity, Table 9 demonstrates the Survival Probabilities of ten patients with Patient Condition (X1) obtained from CNN and distinct risk factors (X2: Blood Pressure, X3: Chronic Obstructive Pulmonary Disease, X4: Blood Sugar, X5: Asthma, X6: Chronic Kidney Disease, X7: Obesity, X8: Acute Respiratory Distress Syndrome, X9: Pulse Oximetry). Column 11 of Table 9 represents the survival probabilities given by a physician by taking account of patients’ data, which is considered as the Expert Opinion (Y(%)), while column 12, column 13, column 14 and column 15 represent the survival probabilities generated from the integrated CNN-BRBES (Non-Trained) (Z(%)), CNN-BRBES (Trained by *fmincon*) ($\alpha$(%)), CNN-BRBES (Trained by BRBAPSO) ($\beta$(%)), and CNN-BRBES (Trained by eBRBaDE) ($\gamma$(%)). Column 16 represents the Outcome. If a patient survived within 14 and 30 days after the initial diagnosis, the outcome is considered as 1. If the patient is deceased, the outcome is considered as 0.

**Table 9:** Survival Probability Assessment of Ten Different Patients

**SL. No**	**X1**	**X2**	**X3**	**X4**	**X5**	**X6**	**X7**	**X8**	**X9**	**Y**	**Z**	$\alpha$	$\beta$	$\gamma$	**Outcome**
1	Non-Critical	Elevated	Mildly Abnormal	Normal	Intermittent	Normal	Level I	Mild	Moderate	85	86	72	81	94	1
2	Non-Critical	Stage 1 Hypertension	Moderately Abnormal	Diabetic	Normal	Normal	Normal	Mild	Moderate	70	74	78	79	83	1
3	Non-Critical	Stage 1 Hypertension	Moderately Abnormal	Normal	Mild Persistent	Moderate	Level I	Mild	Moderate	65	70	74	76	82	1
4	Non-Critical	Elevated	Mildly Abnormal	Diabetic	Intermittent	Mild	Normal	Moderate	Severe	60	62	66	75	83	1
5	Non-Critical	Normal	Normal	Normal	Normal	Normal	Level II	Normal	Moderate	90	93	79	89	97	1
6	Critical	Elevated	Mildly Abnormal	Normal	Intermittent	Moderate	Level I	Severe	Severe	50	52	59	62	69	1
7	Critical	Stage 2 Hypertension	Severely Abnormal	Pre-Diabetic	Moderate Persistent	Severe	Level II	Severe	Moderate	25	29	39	49	61	1
8	Critical	Stage 1 Hypertension	Moderately Abnormal	Normal	Mild Persistent	Moderate	Level I	Severe	Severe	45	42	59	61	54	0
9	Critical	Elevated	Mildly Abnormal	Diabetic	Intermittent	Mild	Normal	Severe	Moderate	55	58	69	67	79	1
10	Critical	Hypertensive Crisis	Moderately Abnormal	Diabetic	Intermittent	Severe	Level II	Severe	Moderate	35	32	47	41	23	0

The receiver operating characteristic (ROC) curve is widely used to measure the reliability and accuracy of the prediction results or analyze the effectiveness of assessment having ordinal or continuous results [39, 40]. Therefore, in this research, it has been considered to test the accuracy of CNN-BRBES against expert opinion.

The accuracy or performance of the CNN-BRBES in assessing the survival probability of a COVID-19 patient can be measured by calculating the area under curve (AUC) [41–43]. The amount of area under curve (AUC) determines the reliability and the accuracy of system-generated results or expert opinions. If the AUC of CNN-BRBES is larger than the expert opinion, it can be inferred that CNN-BRBES produces more accurate and reliable results.

**Fig. 10:**
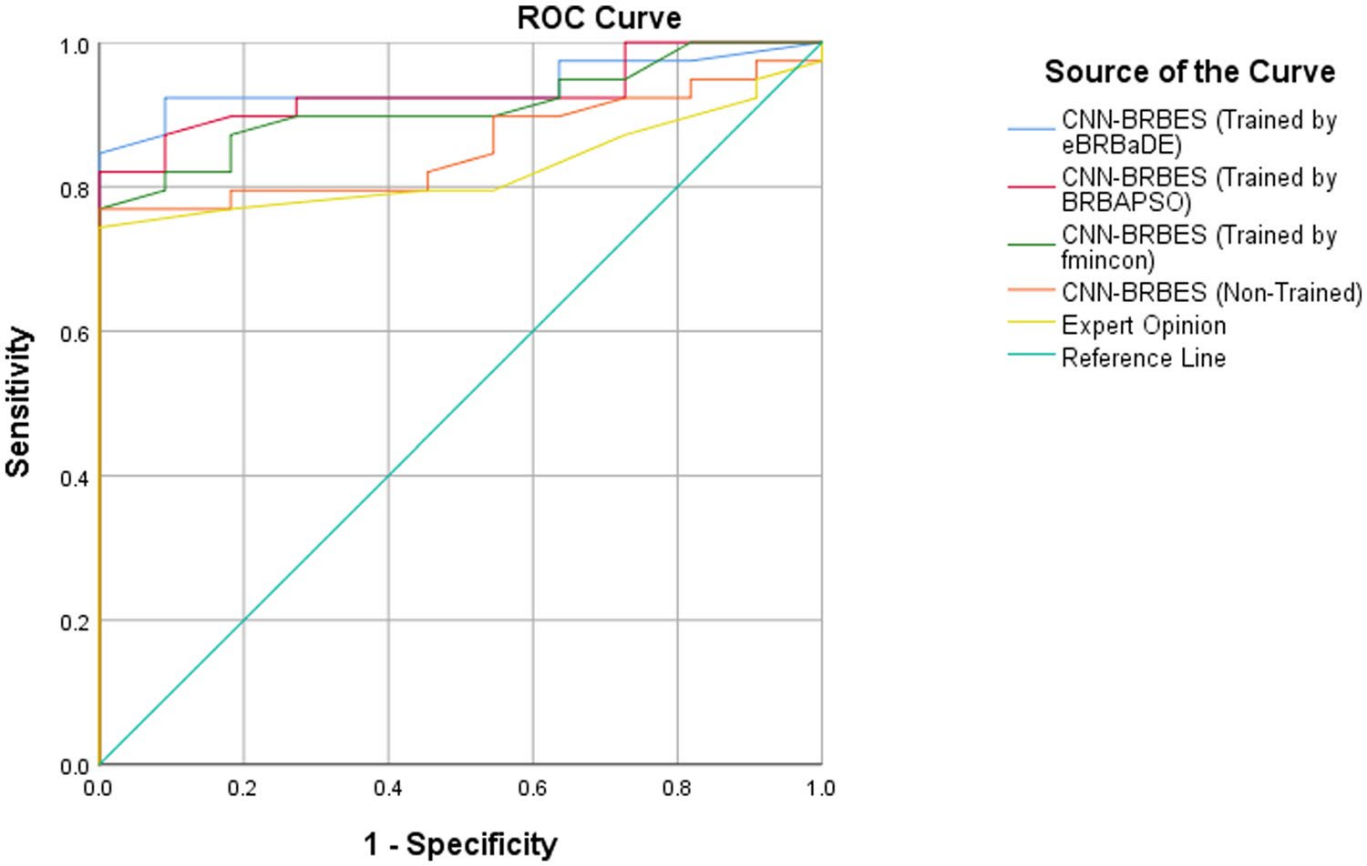
Comparison of Results of CNN-BRBES and Expert Opinion Using ROC Curves

SPSS 25 has been used to generate the ROC curve and calculate the AUC value. Figure 10 shows the ROC curves for the CNN-BRBES (Trained), CNN-BRBES (Non-Trained), and expert opinion, while Table 10 shows the AUC and the confidence interval (CI) for them.

**Table 10:** Comparison of AUC of CNN-BRBES and Expert Opinion

Test Result Variable(s)	AUC	Std. Error	Asymptotic $95\%$	
			Confidence Interval	
			Lower Bound	Upper Bound
CNN-BRBES (Trained by eBRBaDE)	0.938	0.034	0.871	1.000
CNN-BRBES (Trained by BRBAPSO)	0.929	0.036	0.858	0.999
CNN-BRBES (Trained by *fmincon*)	0.910	0.040	0.831	0.989
CNN-BRBES (Non-Trained)	0.855	0.052	0.754	0.957
Expert Opinion	0.825	0.057	0.714	0.937

From the AUC of CNN-BRBES and expert opinion in Table 10, it can be observed that, though the AUC obtained through non-trained CNN-BRBES is not significantly higher than the result generated by expert opinion, the AUC of CNN-BRBES (Trained by eBRBaDE) is significantly greater than the AUC of expert opinion. Besides, for CNN-BRBES (Trained by eBRBaDE), the range of confidence interval is highest, and the standard error is lower than expert opinion. In addition, CNN-BRBES (Trained by eBRBaDE) is performing better because it ensures the balance between exploration and exploitation, which is not the case with CNN-BRBES (Trained by BRBAPSO). CNN-BRBES (Trained by *fmincon*) is not performing better in comparison to others because it uses deterministic optimization approach (SQ). This implies that the results generated by trained CNN-BRBES are better than that of expert opinion, which uses traditional rules without taking account of uncertainty.

## Conclusion

The objective of this research is to propose an integrated CNN-BRBES approach to predict the survival probability of COVID-19 patients. As the CNN part, a customized pre-trained model (VGG19) is employed for COVID-19 patients’ condition assessment that can decide whether or not a patient is a critical COVID-19 patient analyzing chest X-ray image. One of the reasons to use pre-trained models is that in the case of limited data source or amount, it is a proper option to go for the transfer learning approach. Then BRBES carries the remaining responsibility of assessing a patient’s survival probability by analyzing the patients’ information of eight risk factors. The proposed model offers more robustness in results, which are validated by the experts, as it involves both data and knowledge-driven approaches instead of depending on either of these two. Another mentionable point about this research is that a derived dataset (COVID-19-Status) is developed from the most popular dataset in this domain, covid-chestxray-dataset, which is made available for other researchers in GitHub [17].
